# Artificial intelligence in the clinical management and prognostication of mitral regurgitation: a systematic review

**DOI:** 10.1186/s43044-026-00756-1

**Published:** 2026-06-11

**Authors:** Armia Ahmadi-Hadad, Michela Belotti, Assil Zennoud, Boshra Moghimi, Caterina Autuori, Divinie Sumanasekara, Hashini Maneesha Fernando Udiriappuwaduge, Giuseppe Comentale, Emanuele Pilato

**Affiliations:** 1 University of Naples “Federico II” - Napoli, Naples, Italy; 2 University of Campania “Luigi Vanvitelli”- Napoli, Caserta, Italy

**Keywords:** Mitral regurgitation, Artificial intelligence, Machine learning, Deep learning, Echocardiography, Cardiac imaging

## Abstract

**Background:**

Mitral regurgitation (MR), one of the most common valvular heart diseases, poses ongoing challenges in risk stratification and timely intervention. Traditional diagnostic approaches, suffer from interobserver variability. Artificial intelligence (AI) has recently gained traction in cardiology to augment clinical precision. This article reviews the use of AI in the diagnosis, severity assessment, and prognostication of MR, with a focus on performance metrics.

**Methods:**

The search was conducted in PubMed, Embase, and MEDLINE on May 9, 2025. Studies were eligible if they applied AI to MR-related tasks using imaging, ECG, or clinical data. Data extraction focused on dataset characteristics, model architectures, and performance.

**Results:**

A total of eleven studies, comprising 80,915 patients, were included. Among the included studies, six utilized echocardiographic data, two electrocardiography, two clinical biomarkers or structured datasets, and one chest radiography. Algorithms included convolutional neural networks, support vector machines, and ensemble models. Reported AUCs ranged from 0.74 to 0.94. Models based on color Doppler or 3D geometrical mitral features achieved the highest discriminatory performance. Only a minority of studies incorporated external validation or reported clinically actionable thresholds such as PPV. ECG-based models demonstrated high scalability but lower sensitivity. Studies integrating multimodal data yielded promising results.

**Conclusion:**

AI models, especially those trained on echocardiographic imaging, demonstrate strong potential for improving MR evaluation. However, widespread clinical adoption is limited by lack of external validation, and inconsistent outcome reporting. Future work should emphasize model interpretability, multicenter validation, and head-to-head comparisons with expert assessment to bridge the translational gap.

## Introduction

Mitral regurgitation (MR) is among the most common valvular heart diseases in both Europe and the United States [1, 2]. In a large U.S. population-based echocardiographic cohort of 11,911 adults, the prevalence of mitral regurgitation exceeded 10% in individuals aged 75 years and older, highlighting the high burden of disease [1]. Clinical evaluation of MR is limited by substantial interobserver variability in echocardiographic grading, reliance on multiple semi-quantitative parameters, and the increasing use of longitudinal imaging and multimodal data. In this setting, machine learning-based approaches could offer a promising strategy. By utilizing machine learning (ML), the computer receives input data and develops intricate analytical frameworks grounded in a learning paradigm to maximize the precision of forecasts. Deep learning (DL) is a subset of ML based on multi-layered neural networks, which is particularly suited for image-based applications such as medical imaging and echocardiography [3, 4]. Artificial intelligence has established its value in cardiology, extending even to the precise diagnosis of uncommon and rare cardiac diseases [5]. The application of AI in cardiac surgery represents a more recent development. For instance, models trained on CT angiography, contrast-enhanced CT, and non-contrast CT images have shown promise in the diagnosis of aortic aneurysms [6]. Similarly, a model, trained on preoperative variables of patients undergoing aortic arch repair, demonstrated promising performance in predicting postoperative stroke and mortality [7]. The performance of ML models is usually assessed using positive predictive value (PPV, also referred to as precision), negative predictive value (NPV), sensitivity (also referred to as recall), specificity, area under the receiver operating characteristic curve (ROC AUC, measure of overall discriminative ability), and F1-score (calculated as two times the product of precision and recall divided by the sum of precision and recall). This systematic review aims to summarize the current applications of AI in MR, encompassing diagnosis, prognosis of postoperative outcomes, and prediction via regurgitation grading across multiple modalities including echocardiography, ECG, and chest radiography. Moreover, we sought to highlight which of these modalities serves as the most effective substrate for AI model training.

## Materials and methods

The protocol for this systematic review has been registered in PROSPERO under ID: CRD420251114400. This review was conducted in accordance with the Preferred Reporting Items for Systematic Reviews and Meta-Analyses (PRISMA) guidelines [8]. The primary outcome of interest in this investigation is the AI performance score, which quantifies the diagnostic accuracy and prognostic capability of models in the management and prediction of outcomes in MR.

**Fig. 1:**
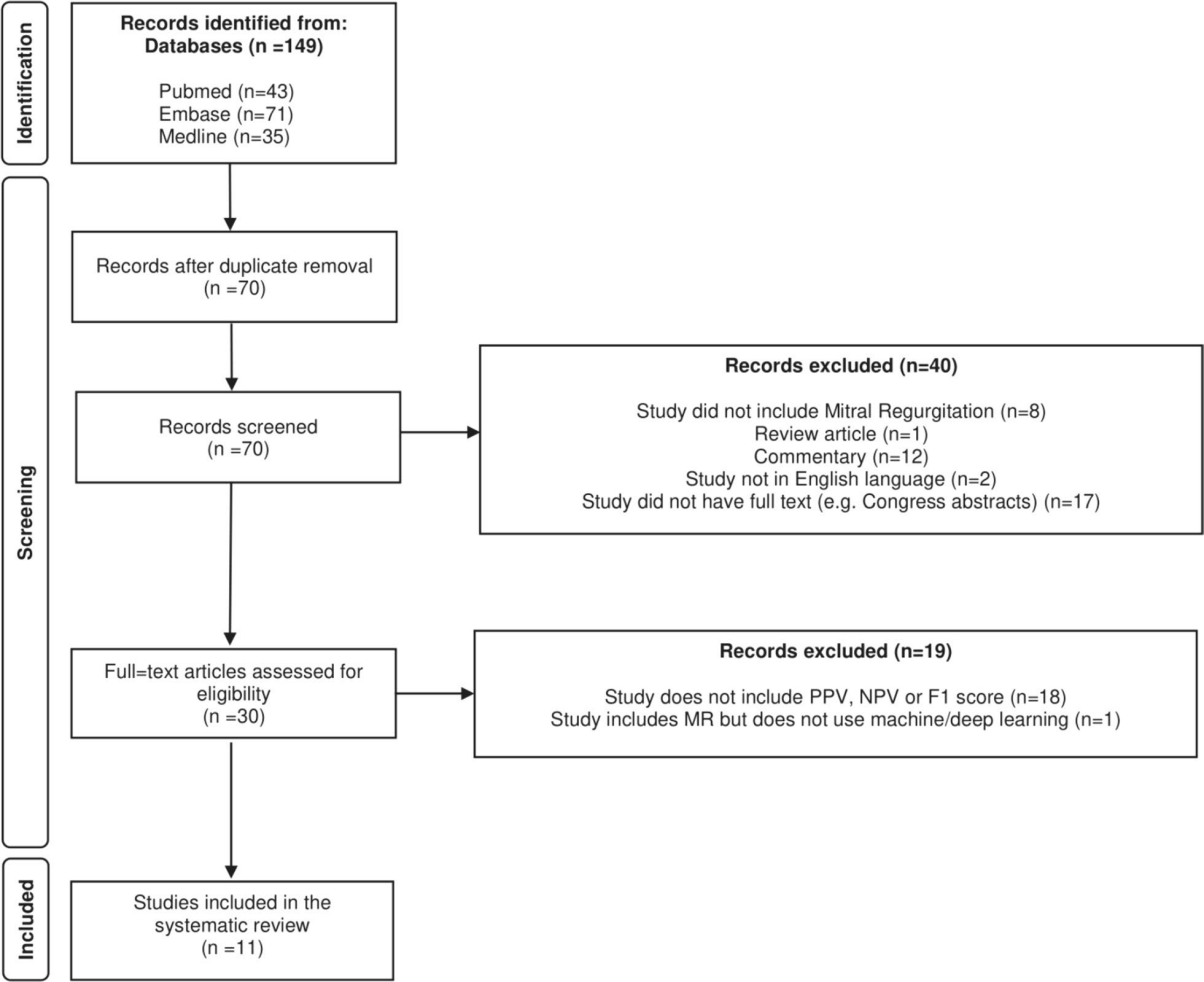
Preferred Reporting Items for Systematic Reviews and Meta-Analyses (PRISMA) diagram

A PRISMA flow diagram was used to document the inclusion and exclusion processes (Fig. 1). The initial search identified 149 results, from which duplicates were excluded. The inclusion and exclusion criteria were then applied through two rounds of screening. As a result, eleven studies were selected for inclusion in the systematic review.

### Search strategy

An extensive literature search was performed on May 9, 2025, across the PubMed, Embase, and MEDLINE (via Ovid) databases, yielding a total of 149 articles. Following the removal of 79 duplicate records, 70 articles were retained for further analysis. The search was conducted without any restrictions on language or publication date. The specific search terms used are detailed in (Table 1).

**Table 1:** Search strategy

Line #	Search	# of results
*PubMed* (ALL—1996 to present)		
Searched on May 9, 2025 No language, article type, or publication date limits		
1	“mitral”[ti]	46,876
2	“regurgitation”[ti]	16,467
3	1 AND 2	8,772
4	(“machine learning”[tw] OR “artificial inte*”[tw])	229,707
5	3 AND 4	43
6	5 NOT “letter”[ti] NOT “comment*”[ti]	43

Line #	Search	# of results
*Embase* (ALL—1946 to present)		
Searched on May 9, 2025 No language, article type, or publication date limits		
1	‘mitral’:ti	61,159
2	‘regurgitation’:ti	24,460
3	1 AND 2	13,281
4	(‘machine learning’:ti,ab,kw,de OR ‘artificial inte*’:ti,ab,kw,de)	299,772
5	3 AND 4	72
6	5 NOT 'letter':ti NOT ‘comment*’:ti	71

Line #	Search	# of results
*Ovid MEDLINE* (ALL—1946 to present)		
Searched on May 9, 2025 No language, article type, or publication date limits		
1	“mitral”.ti	46,905
2	“regurgitation”.ti	16,476
3	1 AND 2	8,781
4	(“machine learning”.tw. OR “artificial inte*”.tw.)	179,988
5	3 AND 4	35
6	5 NOT “letter”.ti. NOT “comment*”.ti	35

The screening of articles was performed by two primary reviewers, MB and AZ in a blinded manner. During the first round, titles and abstracts were reviewed, followed by full-text evaluation in the second round. Any conflicts that arose during the screening process were resolved by a third reviewer, AAH. Ultimately, eleven studies were selected for inclusion in the systematic review (Fig. 1).

### Inclusion and exclusion criteria

The inclusion criteria for this systematic review comprised studies that specifically examined the use of AI models in the management and prognostication of MR. Eligible studies were required to be published in the English language. There were no limitations regarding the classification or severity of MR, allowing the inclusion of both primary and secondary forms of mitral regurgitation. Studies were excluded if they met any of the following criteria: (1) the study did not focus on MR; (2) the study did not utilize machine learning techniques; (3) the study was classified as a review, meta-analysis, case report, letter, editorial, or commentary; (4) the study was not published in English; (5) the full-text was not available (in case of conference abstracts/pre-prints); or (6) other specified reasons for exclusion were identified.

### Data extraction and synthesis

The relevant data from the selected studies were extracted and organized using a structured Excel spreadsheet. The extracted information included study design, control groups, patient demographics (the number of patients, age, and gender), MR severity, and any associated comorbidities. Additional variables considered including the architecture of the model used (CNN-based models, self-supervised learning frameworks, artificial neural networks, and classical machine learning methods such as SVM, LDA, GBM, and XGBoost), along with the key performance indicators for these models such as sensitivity, specificity, F1-score, accuracy, and AUC (area under the curve) of the model.

For each selected paper the year of publication, the location of the study, the name of the leading author, and the journal in which it was published was recorded. In some cases, specific statistical methods used to develop the AI models were also noted to assess their relevance and accuracy in predicting outcomes such as mortality, stroke, and MR severity progression.

### Evaluation metrics

This systematic review employed sensitivity, specificity, positive predictive value (PPV), receiver operating characteristic area under the curve (ROC AUC), and F1-score to assess the efficacy of the models. The ROC AUC serves as a critical measure for binary classification, evaluating the model’s capacity to differentiate between the presence or absence of significant MR. An AUC value closer to 1 reflects optimal model performance, while an AUC of 0.5 suggests random classification. The F1-score, which combines precision (the ratio of true positives to the total predicted positives), was employed to evaluate the model’s performance. This ratio is particularly crucial for accurate MR severity classification and prognostication, ensuring the model’s reliable application in clinical decision-making.

## Results

A comprehensive systematic search was conducted across PubMed (n = 43), Embase (n = 71), and MEDLINE via Ovid (n = 35), yielding a total of 149 records. After removal of duplicates, 70 unique studies remained for screening. Following title and abstract screening, 40 articles were excluded due to not meeting the predefined eligibility criteria. In the second round 30 articles were screened. Subsequently, a set of 11 full-text articles, encompassing a total of 80,915 patients (40,878 for echocardiography-based, 18,372 for ECG-based, 5,270 for the radiograph-based model, and 16,395 for integrated models), was included in the synthesis. An overview of the included studies, model architectures, and patient characteristics are presented in (Tables 2 and 3).

**Table 2:** Characteristics of included studies

Study	Location	sample size	Age	Sex	Raw data	Model architecture	Objective
Vrudhula et al. [9]	USA	58,614 videos from 38,461 patients	NR	Male:57.1% (training), 58.8% (validation), 57.4% (test)	Echo, apical 4 chambers view videos, colored doppler videos	CNN, R (2 + 1) D residual convolutional network	Designing and assessing a DL-based system for the automated recognition of MR in routine TTE examinations
Ueda et al. [19]	Japan	10,367 radiographs on 5,270 patients	67 ± 15 (training), 69 ± 15 (validation), 67 ± 15 (test)	NR	Chest radiographs, echo reports	CNN, ResNet50	Creating a AI system for identifying MR using chest X-ray imaging
Yang et al. 2021 [11]	China	2,766 from 888 patients	69 (59, 79)	Male:585 (65.9)	Colored doppler 2D echo videos	SSL algorithm	Extracting characteristics of the mitral regurgitation jet and left atrial region to support clinicians in assessing the severity of regurgitation
Tsaban et al. [15]	USA	4,019 patients	69.8 ± 15.0	Female: 1,971 (49.0)	ECG, TTE	CNN, ResNet-18	Assessment of all-cause mortality risk in relation to myocardial disease severity, as determined by AI-ECG-based LV dysfunction classification, within the context of MR grading
Moghaddasi et al. [12]	Iran	102 patients	NR	NR	Echo 2D videos	Support vector machine (SVM), linear discrimination analysis (LDA), template matching techniques	Detection of micro-patterns of echo images in order to determine the severity of MR
Zhou et al. [18]	China	706 patients	No TIA: 66 (57–74), TIA: 69 (57–77	NR	Echo, laboratory exams, clinical data	Gradient booster machine (GBM)	Determining key predictors associated with elevated risk of cerebrovascular events and overall mortality
Hausleiter et al. [10]	Germany	4,600 patients	74.1 ± 9.4	Male: 65.4%	Echo, clinical data, biomarkers	XGB boost	Creation and evaluation of an AI–based risk model for forecasting one-year outcomes in individuals with SMR treated with M-TEER
Lv et al. [17]	China	6,704 patients	Isolated MR: 61.33 ± 12.75, MR + TR: 64.62 ± 13.07	Isolated MR: 1,780(60.5), MR + TR: 999(55.3)	Echo, clinical data	CoMT model	Evaluation of patient profiles and prognostic implications in coexisting mitral and tricuspid regurgitation, along with the development of outcome prediction tools
Naser et al. 2023 [16]	USA	18,372 patients	Age ≥ 65 years: for AF 740 (66%) (AI ECG > 29.4%), 213 (48%) (AI ECG ≤ 29.4%); For SR 1355 (54%) (AI ECG > 13.8%), 4186 (29%) (AI ECG ≤ 13.8%)	Female: for AF 360 (32%) (AI ECG > 29.4%), 180 (40%) (AI ECG ≤ 29.4%); For SR 865 (34%) (AI ECG > 13.8%), 6937 (49%) (AI ECG ≤ 13.8%)	ECG	CNN	Recognizing individuals with recently diagnosed atrial fibrillation or sinus rhythm who carry the greatest risk for advancing aSMR
Lachmann et al. [14]	Germany	366 patients	Clauster1: 79.6 ± 5.96, Clauster2: 78.1 ± 6.91, Clauster3: 79.8 ± 8.73, Clauster4: 81.3 ± 6.73	Female: Clauster1: 72 (43.9%), Clauster2: 28 (42.2%), Clauster3: 19 (42.2%), Clauster4: 27 (29.7%)	Echo, right heart catheterisation data	Artificial neural network (ANN)	Evaluation of post-TAVR improvement in non-aortic valvular cardiac injury, assessed in relation to the established stratification framework
Zhang et al. [13]	China	1,427 patients	NR	NR	Echo color doppler images	Mask R-CNN	Development and validation of an AI model algorithm for evaluating MR grades

AF, atrial fibrillation; AI, artificial intelligence; ANN, artificial neural network; AUC, area under the curve; CNN, convolutional neural network; DL, deep learning; GBM, gradient boosting machine; LDA, linear discrimination analysis; M-TEER, mitral transcatheter edge-to-edge repair; MR, mitral regurgitation; NA, not applicable; NPV, negative predictive value; NR, not reported; PPV, positive predictive value; SMR, secondary mitral regurgitation; SR, sinus rhythm; SVM, support vector machine; TAVR, transcatheter aortic valve replacement; TIA, transient ischemic attack; TR, tricuspid regurgitation; TTE, transthoracic echocardiography

**Table 3:** Extended summary of patient characteristics and comorbidities

Study	CAD	Atrial fibrillation	Hypertension	LVEF
Vrudhula et al. [9]	29,661 (44.2%)	19,909 (29.6%)	40,982 (61.0%)	56.56 ± 16.2
Ueda et al. [19]	NR	945 (22%)	NR	6581 (80%): > 50%
Yang et al. [11]	272 (45.9%)	NR	320 (54.1%)	52 [39, 59]
Tsaban et al. [15]	705 (17.6%)	NR	2,134 (53.2%)	56.0 (36.0–65.0)
Moghaddasi et al. [12]	NR	NR	NR	NR
Zhou et al. [18]	NR	NR	590 (83.7%)	NR
Hausleiter et al. [10]	2,677 (58.2%)	2,815 (61.2%)	3,560 (77.4%)	36.0 ± 12.9
Lv et al. [17]	Isolated MR: 1,355 (46.0%), MR + TR: 680 (37.7%)	Isolated MR: 605 (20.6%), MR + TR: 782(43.3%)	Isolated MR: 1,454 (49.4%), MR + TR: 837 (46.4%)	Isolated MR: 52 (37–62), MR + TR: 51 (35.88–60)
Naser et al. [16]	NR	NR	NR	NR
Lachmann et al. [14]	Clauster1: 137 (83.5%), Clauster2: 58 (87.9%), Clauster3: 36 (80.0%), Clauster4: 80 (87.9%)	Clauster1: 33 (20.1%), Clauster2: 25 (37.9%), Clauster3: 34 (75.6%), Clauster4: 69 (75.8%)	Clauster1: 141 (86.0%), Clauster2: 62 (93.9%), Clauster3: 45 (100%), Clauster4: 86 (94.5%)	Clauster1: 57.2 ± 6.36, Clauster2: 55.9 ± 6.47, Clauster3: 42.4 ± 15.7, Clauster4: 47.3 ± 12.2
Zhang et al. [13]	NR	NR	NR	NR

CAD, coronary artery disease; LVEF, left ventricular ejection fraction; MI, myocardial infarction; MR, mitral regurgitation; NR, not reported; TR, tricuspid regurgitation

Among the included studies, various machine learning approaches were employed to predict or classify the severity of MR or its post-intervention prognosis using different data modalities. The studies have been grouped based on the primary data type used to train the model: including echocardiographic measurements (LVEF, LVEDV, left atrial volume, effective regurgitant orifice area, regurgitant volume, vena contracta width, right ventricular dimensions, right atrial area, tricuspid regurgitation grade, systolic pulmonary artery pressure, and speckle-tracking parameters), electrocardiogram (ECG) waveforms, laboratory biomarkers (NT-proBNP, creatinine, C-reactive protein, neutrophil-to-lymphocyte ratio, platelet count, urea, and electrolytes), and structured clinical data (demographics, comorbidities, and surgical history).

Among the AI algorithms trained on echocardiographic data, Vrudhula et al. [9] developed a model trained on four-chamber color Doppler. Hausleiter et al. [10] developed a 1-year mortality prediction model for post endovascular mitral repair. Yang et al. [11] have developed a model based on 2D color Doppler echocardiographic videos. Moghaddasi et al. [12] have developed a machine learning (ML) model for MR severity classification based on TTE (transthoracic echocardiography) data. Zhang et al. [13] developed a deep learning (DL) model to detect and grade MR using color Doppler echocardiography images. Lachmann et al. [14] have developed an artificial neural network (ANN) for long-term recovery prediction of extra-aortic valve cardiac damage including MR and utilized it across different aortic stenosis clusters defined by the authors’ novel risk classification.

Among ECG models Tsaban et al. [15] developed a model for diastolic dysfunction assessment. Naser et al. [16] developed a model capable of identifying patients at risk of developing atrial secondary mitral regurgitation (aSMR) in patients with atrial fibrillation (AF), and in patients with sinus rhythm (SR).

Among models, which have used clinical and laboratory data, Lv et al. [17] developed a 2-year mortality prediction model for patients with concomitant tricuspid and mitral regurgitation. Zhou et al. [18] have developed a multimodal risk stratification model for stroke and all-cause mortality integrating clinical, laboratory, and echocardiographic data.

Ultimately, one MR detection model, developed by Ueda et al. [19] utilized chest radiograph data.

A comprehensive summary of the model performance metrics from the included studies is presented in (Table 4).

**Table 4:** Summary of model performance metrics

Study	PPV	NPV	AUC	Sensitivity	Specificity	F1-score	Other outcomes
Vrudhula et al. [9]	0.805 (moderate MR), 0.626 (severe MR)	0.863 (moderate MR), 0.954 (severe MR)	0.916 (moderate MR), 0.934 (severe MR)	0.807 (moderate MR), 0.626 (severe MR)	NR	0.805 (moderate MR), 0.626 (severe MR)	NA
Ueda et al. [19]	0.68	0.77	0.80	0.71	0. 74	NR	NA
Yang et al. [11]	NR	NR	NR	86.7	90.5	NR	NA
Tsaban et al. [15]	NR	NR	AUC for LVDF: > grade 1: 0.847 > grade 2: 0.911 > grade 3: 0.943	NR	NR	NR	NA
Moghaddasi et al. [12]	NR	NR	NR	99.38	99.63	NR	NA
Zhou et al. [18]	0.78 (due to TIA/stroke), 0.80 (due to all-cause mortality)	NR	0.80 (due to all-cause mortality)	0.79 (due to TIA/stroke), 0,77 (due to all-cause mortality)	NR	0.79 (due to TIA/stroke), 0.78 (due to all-cause mortality)	NA
Hausleiter et al. [10]	0.404	0.889	0.789	0.521	0.834	NR	NA
Lv et al. [17]	NR	NR	0.754	NR	NR	NR	NA
Naser et al. [16]	NA	NA	NA	NA	NA	NA	Incident of aSMR: for AF cohort: HR = 2.20 (95% CI: 1.16–4.17; *P* = 0.02), for SR cohort: HR = 1.44 (95% CI: 1.06–1.96; *P* = 0.02)
Lachmann et al. [14]	NR	NR	NR	Cluster 3: 100, Cluster 4: 85.2	Cluster 3: 0.959, Cluster 4: 0.951	NR	NA
Zhang et al. [13]	NR	NR	NR	0.94 (mild), 0.93 (moderate), 0.92 (severe)	NR	0.94 (mild), 0.91 (moderate), 0.89 (severe)	NA

AUC, area under the curve; LVDF, left ventricular diastolic function; NA, not applicable; NPV, negative predictive value; NR, not reported; PPV, positive predictive value

## Discussion

This systematic review underscores the growing role of artificial intelligence in the diagnosis, prognostic stratification, and clinical decision-making for patients with MR. Among the studies reviewed, echocardiography-based models consistently outperformed those based on ECG or structured clinical data in predictive accuracy. The consistent superior performance of echocardiography-based models likely reflects the rich anatomical and hemodynamic information captured by imaging, which is not available in ECG or structured clinical datasets. However, widespread clinical implementation remains limited due to a lack of external validation in some studies, absence of standardized clinical thresholds, and variability in outcome definitions.

### Echocardiographic models

Echocardiographic data formed the foundation of most high-performing models.

Six studies trained AI algorithms using echocardiographic data. In 2024, Vrudhula and colleagues [9] focused on MR diagnosis, introducing and validating a fully automated framework capable of identifying and quantifying MR from apical four-chamber color Doppler echocardiographic recordings. Training was performed on over 58,000 studies from Cedars-Sinai and the model was externally tested on 915 studies from Stanford. Ultimately, a high performance was achieved with AUC up to 0.969 for severe MR. The approach enables accurate, scalable MR screening from full TTE across institutions [9]. According to current ACC/AHA valvular heart disease guidelines, echocardiography remains the primary modality for MR quantification; however, these assessments are subject to considerable interobserver variability, particularly during steps such as view and frame selection, color Doppler jet interpretation, proximal isovelocity surface area, radius measurement, and vena contracta tracing. AI-based frameworks, such as those developed by Vrudhula et al., may address this limitation by providing reproducible, automated quantification across large datasets [20] .

Hausleiter et al. [10] on the other hand focused on MR prognosis, developing a supervised learning algorithm (EuroSMR score) based on XGBoost (extreme gradient boost) that integrates 18 structured clinical, echocardiographic and laboratory parameters to predict 1-year mortality following mitral transcatheter edge-to-edge repair in patients with secondary mitral regurgitation achieving an AUC of 0.789 and improving prognostic accuracy compared to traditional risk scores. While current guideline-endorsed risk models (e.g., EuroSCORE II, STS score) provide general perioperative risk assessment, they are not tailored to MR-specific prognostication. AI-enhanced models such as the EuroSMR score extend beyond guideline recommendations by integrating multimodal data, potentially offering more individualized predictions of post-TEER outcomes.

Yang et al. [11] developed a self-supervised learning algorithm (CD-SSL) capable of automatically extracting quantitative indices from 2D color Doppler echocardiographic videos, thereby improving diagnostic accuracy in MR severity classification compared to visual assessment alone. Moghaddasi et al. [12] developed an ML model for MR severity classification based exclusively on TTE video data. The dataset included 139 patients across four MR severity stages. Feature extraction was performed using Extensive Local Binary Pattern (ELBP) and Extensive Volume Local Binary Pattern (EVLBP) applied to multiple echocardiographic views and synchronized ECG frames. Dimensionality reduction was implemented via Principal Component Analysis (PCA). Several classifiers were evaluated, with the Support Vector Machine (SVM) with Radial Basis Function kernel (RBF kernel) yielding the best performance, achieving an overall sensitivity up to 99.38%, specificity up to 99.63%. Zhang et al. [13] developed an automated model based on the Mask R-CNN architecture to detect and grade MR using color doppler echocardiography images. Trained on 1,132 studies from Shandong Provincial Third Hospital and externally validated on 295 studies from a second tertiary center, the model demonstrated strong performance as a F1-score of 0.94 for mild MR and an accuracy up to 0.91 for severe MR. Lachmann et al. [14] on the other hand, focused on MR prognosis. They have applied an AI-enabled phenotyping framework to stratify patients with severe aortic stenosis undergoing transcatheter aortic valve replacement, using preprocedural echocardiographic and right heart catheterization data. Trained on 366 patients from two German centers, the model identified prognostically distinct clusters and predicted long-term recovery of extra-aortic valve cardiac damage, with specific consideration of mitral regurgitation. Across echocardiography-based models, AUCs ranged from 0.789 to 0.916. However, the reported sensitivity of 100% for cluster 3 may indicate potential overfitting, particularly in light of the small sample size and absence of external validation, undermining the reliability of the model’s performance for this cluster.

It is of note that while these models can significantly enhance diagnostic and prognostic capabilities in MR, translating these findings into clinical practice requires standardization, external validation, and careful consideration of patient-specific factors such as age, comorbidities, and MR etiology.

### ECG models

Two studies utilized ECG signals to develop prediction models. ECG-based models hold substantial promise for prognostication in MR, as AI can discern pathological patterns within waveforms that may otherwise appear physiologically normal. However, these models tended to show greater variability in sensitivity. According to current ACC/AHA valvular guidelines, the role of ECG in MR management is limited to identifying rhythm disturbances (e.g., atrial fibrillation) or indirect evidence of chamber enlargement, without any role in MR quantification or prognostication [20]. AI-enhanced ECG models may extend this role by uncovering subclinical electrophysiological patterns that carry prognostic value.

Tsaban et al. [15] have focused on prognostic aspects of MR, integrating ECG data with echocardiographic parameters, including left ventricular diastolic function (LVDF) indices such as mitral inflow and annular tissue Doppler velocities. In patients with significant Mitral regurgitation, impairment of left ventricular function may reflect underlying remodeling processes that develop before overt symptoms or structural abnormalities become apparent. Detecting such dysfunction may therefore help identify individuals at increased risk of adverse clinical progression and death. This multimodal ECG/echocardiography-based model demonstrated promising results and scalability due to low acquisition costs and wide availability. Their model using ResNet-18 has accurately assessed diastolic function in patients with significant MR. In a cohort of 4019 patients, higher AI-derived diastolic dysfunction grades were strongly associated with increased mortality, independent of echocardiography findings. The model achieved AUCs up to 0.943 showing potential as non-invasive prognostic tool in MR management. This model underscores the potential of AI-ECG to provide prognostic insights non-invasively, identifying patients at higher risk of adverse outcomes and potentially enabling early intervention, particularly in settings where echocardiography may be limited or less accessible.

Naser et al. [16] demonstrated that an AI-enabled ECG model based on CNN can identify patients at risk of developing aSMR. In a cohort of 18,372 individuals with or without AF, higher AI-derived AF probabilities were independently associated with incident aSMR, left atrial enlargement and diastolic dysfunction. These findings highlight the value of AI-ECG in detecting subtle electrophysiological changes that precede clinical manifestations, suggesting a role for AI in early risk stratification and guiding monitoring strategies for patients susceptible to valvular remodeling and atrial myopathy.

Overall, ECG-based models demonstrate that AI can complement imaging-based approaches, providing prognostic information from widely available and low-cost data, though variability in performance underscores the need for validation across diverse patient populations. Future ML models may also explore the feasibility of grading MR directly from ECG data; if achievable, this approach could eventually be adapted for use in wearable devices such as smartwatches.

### Models based on clinical data and biomarkers

While current guidelines highlight the importance of clinical and laboratory parameters for risk stratification in MR, imaging remains central to severity assessment and intervention timing [20]. Provided that they are adequately trained and externally validated, AI-based clinical and biomarker models hold potential to complement these guideline-directed assessments by integrating multiple patient variables to improve prognostic precision, particularly in borderline or complex cases.

The included biomarker- and clinical data–based models demonstrated moderate predictive accuracy, however offered advantages in interpretability and feasibility for integration into existing electronic health records. Nonetheless, their lack of imaging input limited their precision in risk stratification, particularly for borderline cases, and their sample heterogeneity contributed to weakening the predictive power [17, 18]. Despite moderate accuracy, these models provide clinically actionable insights due to their interpretability, allowing clinicians to understand which variables drive predictions and to potentially guide individualized care in a practical, real-world setting.

Lv et al. [17] focused on MR prognosis, centering on patients with combined mitral and tricuspid regurgitation, demonstrating that this group had significantly worse clinical profiles and outcomes compared to isolated valve regurgitation. They developed and externally validated a dedicated prognostic model (CoMT) for 2-year mortality prediction, reporting an AUC of 0.754. This prognostic model highlights the importance of considering comorbid valvular lesions when evaluating patient risk and demonstrates how AI can integrate multiple clinical parameters to inform long-term outcome predictions.

Zhou et al. [18] conducted a retrospective analysis of a cohort of 706 patients with MR, integrating clinical (age and blood pressure), laboratory (urea, platelet count, albumin, and, sodium), echocardiographic (LVEF, MR regurgitant volume, end-systolic left atrium dimension, velocity–time integral, and effective regurgitant orifice), and electrocardiographic (average P-wave duration) data. P-wave indices, may serve as valuable non-invasive markers of MR severity, as chronic regurgitant volume overload promotes progressive left atrial remodeling, which is reflected by abnormalities in P-wave dispersion, terminal force, and duration.

Zhou et al. [18] developed and validated this risk stratification model based on Gradient Boosting Machine (GBM), which demonstrated high predictive accuracy for both cerebrovascular events (AUC:0.8084) and all-cause mortality (AUC:0.7962) outperforming conventional models and other ML algorithms. These findings illustrate the ability of AI-based clinical models to support risk stratification for multiple adverse outcomes, potentially aiding in the early identification of high-risk patients even in the absence of imaging data.

### Chest radiograph model

Ueda et al. focused on MR diagnosis, developing and validating an AI model capable of detecting MR from chest radiographs, achieving an AUC of 0.80 in both the validation and test sets. By using labeled radiographic images linked to echocardiographic findings, the model was trained to classify the presence or absence of MR. The ultimate goal was to provide an objective, accessible, and non-invasive diagnostic tool that could support clinical decision-making, especially in settings where echocardiography is unavailable and impractical [19]. While current valvular heart disease guidelines do not recommend chest radiography for MR quantification and emphasize echocardiography as the primary diagnostic modality, AI-based chest radiograph models could potentially serve as a complementary diagnostic tool, particularly in cases of functional MR. The moderate PPV of 68% reported by Ueda et al. may be partly attributable to the design of the model’s training dataset, which did not distinguish between different MR etiologies.

Across the entirety of the selected studies, several models achieved strong performance metrics, with AUC values ranging from 0.74 to 0.94. However, not all the studies validated their results on external cohorts, and only a minority reported clinically actionable thresholds or incorporated models into clinical workflows. This limits immediate clinical translatability.

Models incorporating echocardiographic imaging demonstrate strong diagnostic and prognostic potential. However, multimodal approaches, particularly those integrating echocardiography and ECG, offer significant promise for enhanced predictive accuracy by capturing complementary anatomical and physiological information. Such integration can potentially allow clinicians to identify high-risk patients earlier and tailor monitoring or interventions more effectively, illustrating how AI may complement rather than replace existing clinical assessments. Nevertheless, a general trend demonstrates that multimodal and image-based models outperform text-based or single-signal models in predictive accuracy, while clinical models retain value in real-world settings due to generalizability and lower technical barriers.

It is important to note that AI applications in MR should be carefully adopted by counting for characteristics of the training population (e.g., age, comorbidities) and the validation status of the model, to ensure safe and meaningful translation into patient care.

On average, most patients included in the training datasets of included AI models were sexagenarians, with mean ages ranging from 61.3 to 69.8 years, suggesting that model performance is likely optimal within this age range. A few models, however, incorporated older populations: for example, the mitral transcatheter edge-to-edge repair prognostic model by Hausleiter et al. was trained on patients with a mean age of 74.1 years, while the post-transcatheter aortic valve replacement model by Lachmann et al. included patients aged 78.1 to 79.8 across different clusters. Notably, no AI model to date has been specifically developed for younger patients in their 40s or 50s, highlighting an important area for future research.

In terms of applicability of the models with respect to patient comorbidities, coronary artery disease prevalence ranged from 44.2% to 58.2% in most studies. However, in the mortality prediction model of Tsaban et al., patients with coronary artery disease constituted only 17.6% of the dataset, making it potentially more suitable for prognostication in populations with fewer prior coronary issues. By contrast, the dataset of the post-transcatheter aortic valve replacement model by Lachmann et al. included a markedly higher proportion of patients with coronary artery disease, ranging from 80.0% to 87.9%. AF prevalence among patient cohorts in the included studies was generally in the low to moderate range of 20.1%–43.3%. The cohort of Hausleiter et al., however, had 61.2% AF, which is expected given the model’s focus on SMR patients, where AF is a major contributor to functional MR. Likewise, clusters 3 and 4 of Lachmann et al. included 75.6% and 75.8% AF patients, respectively. Hypertension prevalence in the training datasets of the models ranged from 46.4% to 100%, a rate consistent with the advanced age of the included patient populations. LVEF among the included patients ranged from 42.4% to 57.2% in most studies, with the exception of Hausleiter et al., whose cohort had an average LVEF of 36.0%, making it a particularly suitable model for SMR patients with more severe impairment of systolic function.

Further research is also required to ensure external validation, and to compare the model’s accuracy against current MR guidelines.

## Risk of bias assessment

Risk of bias was assessed using the Risk Of Bias In Non-randomized Studies of Interventions (ROBINS-I) tool [21]. Overall, the majority of included studies were judged to have a low-to-moderate risk of bias. Confounding, and selection of reported results were the most frequently identified domains contributing to increased risk of bias across studies. Studies conducted by Zhou et al. and Lachmann et al. were assessed as having an overall serious risk of bias, primarily driven by serious risk in the confounding domain, and the study by Tsaban et al. was assessed as having overall serious risk of bias driven by confounding, measurement of outcomes, and selection of reported results. The results of the assessment are summarized in (Table 5).

**Table 5:** Risk of bias assessment of included studies using the Risk of Bias in Non-randomized Studies of Interventions (ROBINS-I) tool

Study	Confounding	Classification of interventions	Selection of participants	Deviations from intended interventions	Missing data	Measurement of outcomes	Selection of reported results	overall risk of bias
Vrudhula et al. [9]	Moderate	Moderate	Low	Low	Moderate	Moderate	Moderate	Moderate
Ueda et al. [19]	Moderate	Low	Moderate	Low	Low	Low	Low	Moderate
Yang et al. [11]	Moderate	Low	Moderate	Low	Moderate	Moderate	Moderate	Moderate
Tsaban et al. [15]	Serious	Moderate	Moderate	Low	Moderate	Serious	Serious	Serious
Moghaddasi et al. 2016 [12]	Moderate	Low	Low	Low	Low	Low	Low	Moderate
Zhou et al. 2023 [18]	Serious	Moderate	Moderate	Low	Low	Low	Low	Serious
Hausleiter et al. 2024 [10]	Moderate	Low	Low	Moderate	Moderate	Moderate	Moderate	Moderate
Lv et al. 2025 [17]	Moderate	Low	Moderate	Low	Moderate	Low	Moderate	Moderate
Naser et al. 2023 [16]	Moderate	Low	Moderate	Low	Low	Low	Low	Moderate
Lachmann et al. 2022 [14]	Serious	Low	Low	Moderate	Moderate	Low	Moderate	Serious
Zhang et al. 2021 [13]	Low	Low	Low	Low	Low	Low	Low	Low

## Limitations

The number of eligible studies was limited, and many were single-center investigations with retrospective designs, which reduces the generalizability of their findings. The limited number of included studies, heterogeneity in study objectives (diagnostic, prognostic, or grading), inconsistent reporting of performance metrics and 95% confidence intervals, and lack of information on the number of patients included in the training datasets prevented the conduction of a clinically meaningful meta-analysis. Moreover, the absence of external validation in most models raises concerns about overfitting and restricts the applicability of results to broader clinical settings.

The included studies often lacked standardized reporting of key model characteristics and metrics, such as training/test data separation, calibration methods, decision thresholds, PPV, and NPV. Although the Matthews Correlation Coefficient is generally preferred for highly imbalanced datasets, most of the included studies have not reported this parameter. Moreover, in many cases the comorbidities of patients in the training datasets were not reported comprehensively, limiting the ability to determine the populations in which these models may perform optimally. This limited our ability to assess model robustness and clinical utility. Publication bias may have influenced the study pool, as models with suboptimal performance may not have been reported or published. Furthermore, wide variability in sample size (ranging from 139 to over 58,614 patients) may also introduce selection bias. Moreover, integrated models were generally trained on smaller cohorts (mean: 3,279 patients per study), likely reflecting the increased complexity of harmonizing heterogeneous data sources across imaging, electrocardiographic, and clinical domains. These factors might limit the establishment of reliable performance benchmarks. Finally, while risk of bias was systematically assessed using ROBINS-I framework, some domains, such as confounding and outcome measurement, remain difficult to evaluate in AI studies due to inconsistent documentation. Together, these limitations highlight the need for more rigorously designed, prospectively validated, and transparently reported studies in the context of AI use in MR.

## Conclusions

Machine learning models demonstrate substantial potential for improving the diagnosis, severity classification, prognostic stratification, and clinical decision-making in patients with mitral regurgitation. Across the reviewed studies, echocardiography-based and multimodal models consistently achieved the strongest predictive performance, particularly when integrating anatomical, hemodynamic, electrocardiographic, and clinical variables, while ECG-based approaches also demonstrated promising prognostic utility. Future research should focus on prospective multicenter validation, and direct benchmarking against guideline-directed assessment strategies to facilitate broader clinical applicability. Additional efforts are warranted to establish clinically actionable thresholds, and optimize model performance across diverse patient populations and MR etiologies.

## Data Availability

The extracted raw data supporting this systematic review will be made available upon request.

## Abbreviations

AF: Atrial fibrillation
AI: Artificial intelligence
ANN: Artificial neural network
aSMR: Atrial secondary mitral regurgitation
AUC: Area under the curve
CABG: Coronary artery bypass grafting
CD-SLL: Contrastive deep semi-supervised learning
CNN-LSTM: Convolutional neural network-long short-term memory
DL: Deep learning
ELBP: Extensive local binary pattern
EVLBP: Extensive volume local binary pattern
GBM: Gradient boosting machine
HR: Hazard ratio
LDA: Linear discrimination analysis
LV: Left ventricular
LVEDV: Left ventricular end-diastolic volume
LVEF: Left ventricular ejection fraction
MI: Myocardial infarction
ML: Machine learning
MR: Mitral regurgitation
NPV: Negative predictive value
PCA: Principal component analysis
PPV: Positive predictive value
RBF kernel: Radial basis function kernel
SMR: Secondary mitral regurgitation
SR: Sinus rhythm
SVM: Support vector machine
TTE: Transthoracic echocardiography
TR: Tricuspid regurgitation
XGBoost: Extreme gradient boost
